# Efficient Generation of Megakaryocyte Progenitors and Platelets From HSPCs via JAK2/STAT3 Signaling

**DOI:** 10.1002/advs.202500612

**Published:** 2025-04-29

**Authors:** Huicong Liu, Lingna Wang, Jiaqing Liu, Haitao Yuan, Kaiqing Zhang, Yun Qiu, Fangfang Zhu

**Affiliations:** ^1^ School of Biomedical Engineering Shanghai Jiao Tong University Shanghai 200030 China

**Keywords:** GABA, HDAC, HES7, JAK/STAT, megakaryocyte progenitor, platelet

## Abstract

The supply of platelets for clinical transfusion is often insufficient to meet growing demand. Platelet regeneration from stem cells offers a potential solution to reduce reliance on donor‐based transfusions. However, the current differentiation efficiency is suboptimal. A novel approach is presented that significantly enhances platelet yield from hematopoietic stem and progenitor cells (HSPCs) by increasing the production of megakaryocyte progenitors (MkPs) and mature megakaryocytes (MKs). This method employs the overexpression of HES7 combined with the HDAC inhibitor and GABA agonist (collectively termed the VGM cocktail). The VGM cocktail induces MkP production with an efficiency of up to 90%, validated across HSPCs from various donors. These MkPs exhibit extended proliferative capacity, remaining viable for up to 51 days in prolonged culture, and show enhanced maturation into MKs. This differentiation system effectively replicates in vivo thrombocytopoiesis, as evidenced by polyploidization, long protrusions, and proplatelet formation. Transfusion of VGM‐induced MkPs into thrombocytopenic mice results in the release of platelets into circulation. Mechanistic investigation identifies the JAK2/STAT3 signaling pathway as critical in promoting megakaryopoiesis within this system. Therefore, this study demonstrates that the VGM cocktail facilitates enhanced platelet production by promoting MkP generation, offering a promising strategy for in vitro platelet regeneration for clinical applications.

## Graphical abstract

This research presents a novel strategy employing the VGM cocktail, which combines HES7 overexpression with HDAC inhibitor and GABA, to enhance platelet production from hematopoietic stem and progenitor cells by promoting megakaryocyte progenitor (MkP) generation. This method increases MkP yield up to 90%, extends proliferation, and facilitates maturation. The JAK2/STAT3 pathway is identified as a key regulator in this process.

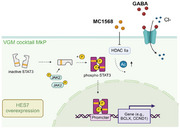

## 1 Introduction

The increasing demand for platelet transfusions has underscored the critical need for efficient in vitro platelet production. Despite extensive research focused on generating large‐scale platelets from hematopoietic stem and progenitor cells (HSPCs) and pluripotent stem cells (PSCs), the yield remains insufficient for clinical applications.^[^ ^1^ ^]^

Megakaryocytes (MKs), the precursors to platelets, arise from hematopoietic stem cells (HSCs) through a well‐orchestrated differentiation process. To meet clinical requirements, large numbers of MKs biased toward thrombopoiesis are essential. However, in vitro‐generated MKs often display injury and asynchronous differentiation.^[^ ^2^ ^]^ Establishing the proliferative capacity of megakaryocyte progenitors (MkPs) could provide a solution by sustaining platelet production over time. MkPs were first defined in 1982 by Paulus et al.,^[^ ^3^ ^]^ and adult human MkPs in the bone marrow were later identified by Miyawaki et al. in 2017, characterized by the CD34^+^CD38^+^CD45RA^−^IL3‐Rα^dim^CD41^+^ phenotype,^[^ ^4^ ^]^ demonstrating robust megakaryocyte‐specific lineage potential. Subsequent studies, including those by Pick et al.,^[^ ^5^ ^]^ Jiang et al.,^[^ ^6^ ^]^ and Nakamura et al.,^[^ ^7^ ^]^ have explored methods to generate clonogenic MkPs and immortalized MkP cell line (imMKCL) from human PSCs. However, these methods either showed very limited proliferation of MkPs^[^ ^5^, ^6^ ^]^ or had to reply on the (proto‐)oncogens c‐MYC, BMI1, and BCL‐XL.^[^ ^7^ ^]^

MK lineage commitment from HSCs is orchestrated by the precise coordination of several levels of modulators, including transcription factors (TFs), epigenetic regulators, and signaling pathway molecules. TFs such as FLI1, GATA1, GATA2^[^ ^8^ ^]^ have been reported by numerous studies to play important roles in MK differentiation. Notably, transient expression of c‐MYC has been identified as crucial for MK maturation into platelets from human PSCs,^[^ ^9^ ^]^ which was then used to establish imMKCL for platelet generation.^[^ ^7^ ^]^ Our previous research, by combining the *Gene Expression Commons (GEXC)* microarray platform and CRISPR/Cas9 technology, has identified several novel TF regulators of MkP differentiation.^[^ ^10^ ^]^ Epigenetic regulations, through histone deacetylases (HDACs) for example, act at different levels to influence MK differentiation.^[^ ^11^ ^]^ For instance, HDAC1 and HDAC2 interact with FOG‐1 to activate the GATA switch to promote MK terminal differentiation,^[^ ^11^ ^]^ and the HDAC inhibitor trichostatin A was reported to inhibit GATA1 expression and thereby delay MK maturation,^[^ ^12^ ^]^ while paradoxically, HDAC inhibition has been shown to enhance MkP differentiation,^[^ ^10^ ^]^ indicating the underlying complex regulation at different stages of MK lineage commitment. Additionally, γ‐Aminobutyric acid (GABA) signaling, primarily known for its role in neurogenesis, has been implicated in hematopoiesis and HSPC engraftment through the receptor GABRR1 on HSPCs,^[^ ^13^ ^]^ and our previous findings also confirmed the positive influence of GABA on platelet generation.^[^ ^14^ ^]^

The Janus kinase/signal transducer and activator of transcription (JAK/STAT) signaling pathway, which mediates over 50 cytokine signals,^[^ ^15^ ^]^ is another critical regulator in hematopoiesis.^[^ ^16^ ^]^ JAK2, in particular, is a key mediator in myeloid lineage differentiation by interacting with receptors like EPOR and TPOR/MPL, among others.^[^ ^17^ ^]^ Upon cytokine binding to its receptor, the associated JAKs are activated, enabling the phosphorylation, dimerization, and nuclear translocation of STAT proteins, which then drive the expression of cytokine‐responsive genes.^[^ ^18^ ^]^ This pathway's role in hematopoietic lineage determination underscores its potential impact on MK differentiation and platelet production.

Given these insights, our study aims to develop an efficient method for platelet production from HSPCs by combining these important regulators. We identified HES7 as the key TF, among other TFs tested, that drives MK lineage commitment and demonstrated the synergistic effect of the combination of HES7 with the HDAC inhibitor MC1568 and GABA. Based on this, we development a novel procedure consisting of MC1568, GABA, as well as HES7 overexpression, termed the VGM cocktail, that significantly increases MkP differentiation efficiency to 90%, and enhances the proliferative capacity of MkPs. This approach enabled the production of a substantial amount of platelets both in vitro and in vivo. Furthermore, we uncovered the crucial role of the JAK2/STAT3 signaling pathway in mediating these effects. Therefore, our findings offer a promising strategy for clinical‐scale platelet regeneration.

## 2 Result

### 2.1 Enhanced MkP Differentiation via Transcription Factor Overexpression and Small Molecule Combination

**Figure 1:**
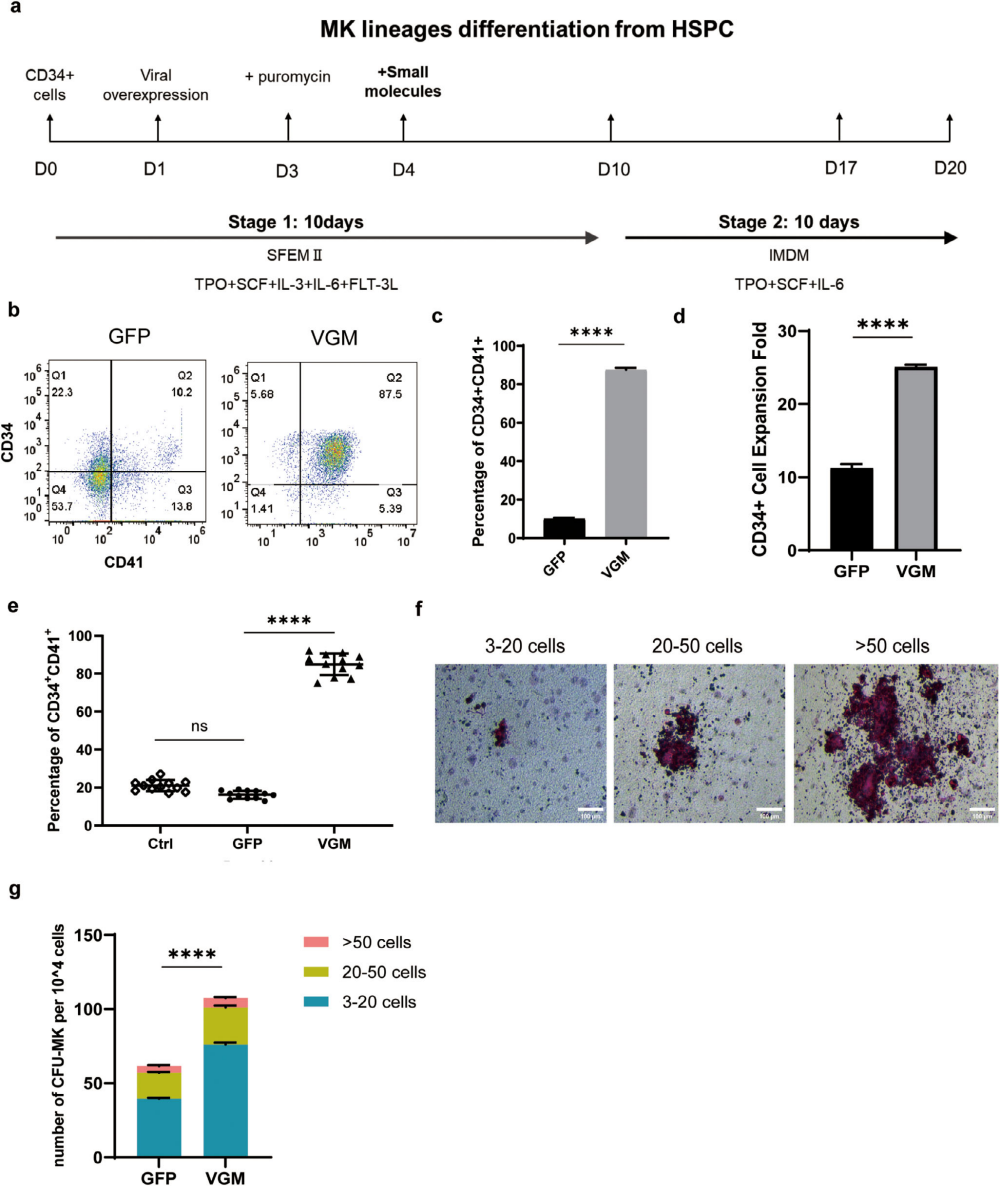
VGM cocktail significantly improved MkP differentiation in vitro. a) Schematic outlining of the in vitro differentiation protocol. b) Representative dot plots from flow cytometric analysis showing the expression of CD34 and CD41 on cells in GFP and VGM groups on differentiation day 10. c) Quantification of the percentage of CD34^+^CD41^+^ cells in (b). d) Quantitative analysis of CD34^+^CD41^+^ cell expansion on day 10. e) Statistical analysis of CD34^+^CD41^+^ cell populations from 12 different batches of cells treated with VGM. (f) CFU‐MK derived from MkP were identified through CD41 staining and classified into the following categories: small (3–20 cells), medium (20–50 cells), and large (≥ 50 cells), with scale bars set at 100 µm. g) The absolute number of CFU‐MK was quantified under different treatment conditions. Data are means ± SD of biologically independent samples. Statistical significance was calculated using a two‐tailed *t*‐test and an ordinary one‐way ANOVA. The statistical significance indicated in the figures was assigned as not significant (ns) *p* > 0.05; ^****^ *p* < 0.0001.

In this study, we sought to identify methods to improve the efficiency of human MkP and platelet differentiation. We utilized a differentiation protocol adapted from Zhu et al.^[^ ^14^ ^]^ (**Figure** **1**a).

We first assessed the impact of overexpressing individual TFs on MkP differentiation, including HES7, FLI1, MXD3, FOXB1, HOXA9, and C‐MYC. Unipotent MkPs, originally characterized by Miyawaki et al., are crucial for platelet generation due to their robust megakaryocyte‐specific lineage potential.^[^ ^4^ ^]^ After 10 days of differentiation, HES7 overexpression resulted in the highest efficiency (25.13% ± 2.60%). Interestingly, co‐overexpression of HES7 with any of the other five TFs did not further enhance differentiation efficiency (Figure S1a,b, Supporting Information). Similarly, combining HES7 with multiple other TFs or various combinations among the other TFs also failed to yield significant improvements (Figure S1c–f, Supporting Information). These findings demonstrate that HES7 is the most effective TF for promoting MkP differentiation among those tested.

Given the presence of GABA receptors on HSCs and MkPs, which form the GABA‐GABRR1 axis to facilitate MkP generation,^[^ ^14^ ^]^ and the known inhibitory role of HDACs on MkP production,^[^ ^10^ ^]^ we explored whether combining GABA and HDAC inhibitors (HDACi) with HES7 overexpression could synergistically enhance MkP production. Optimal concentrations were identified as 80 mm GABA for GABA agonist and 5 µm MC1568 for HDACi, inducing 13.65% ± 0.21% and 29.05% ± 2.05% MkP production, respectively (Figure S2a–f, Supporting Information). CD34^+^ cells overexpressing HES7 were then cultured with GABA and MC1568 (VGM cocktail) from day 4 to day 10. By day 10, ≈90% (87.53% ± 1.11% vs 10.07% ± 0.45% in GFP group) of the cells had differentiated into CD34^+^CD41^+^ MkPs (Figure 1b,c). We also counted cells differentiated up to day 10 and found the CD34^+^ cells treated with VGM and GFP achieved expansion folds of ≈25.1 and ≈11.4, respectively (Figure 1d). This high efficiency was consistent across different batches (84.92% ± 5.65% vs 16.23% ± 2.06% in GFP group), establishing the VGM cocktail as the most effective condition for inducing MkP differentiation (Figure 1e).

To further validate the identity of VGM‐treated MkPs, we conducted a Colony Forming Unit (CFU)‐MK assay. The VGM‐treated group produced a higher number of MK colonies than the GFP‐treated group (Figure 1f,g), confirming that VGM treatment enhanced the differentiation of HSPCs into MkPs.

### 2.2 VGM Cocktail‐Induced MkPs Exhibit Enhanced Proliferative Capacity

**Figure 2:**
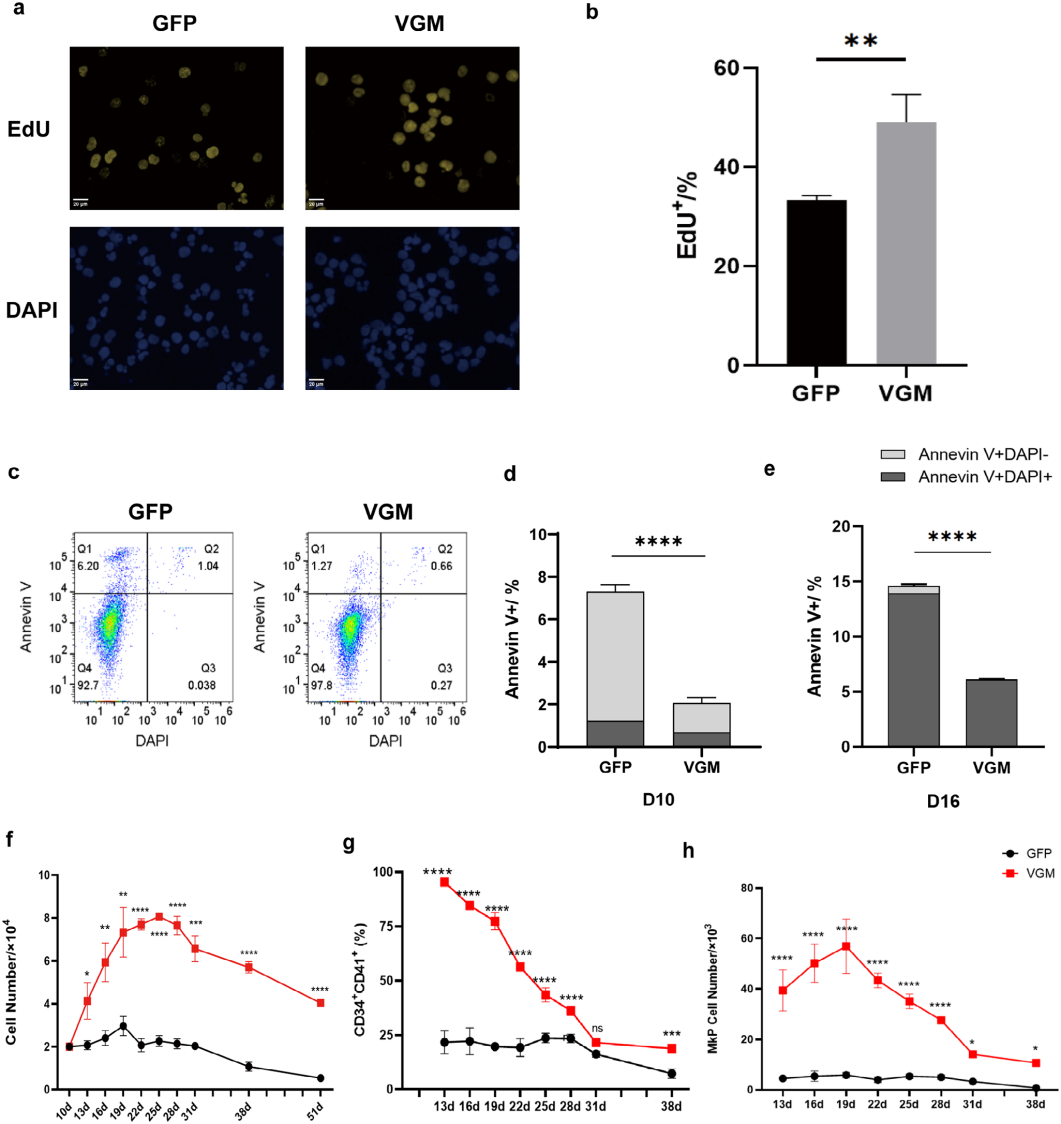
VGM cocktail promoted the long‐term proliferative capacity of MkPs. a) Representative images of EdU (yellow) and DAPI labeled nuclei (blue) of cells in GFP and VGM groups on differentiation day 10. Scale bar, 20 µm. b) Quantification of EdU positive cells in (a). *n* = 3 biological replicates. Four images per n. At least 5000 cells per n were calculated. c) Flow cytometry analysis of apoptotic MkP populations at day 10 from GFP‐ and VGM‐treated cells, based on Annexin V and DAPI staining. d,e) Statistical analysis of apoptotic cell populations in GFP and VGM groups on differentiation day 10 d) and day 16 e), based on Annexin V and DAPI staining. f) Summary of the routine cell counting in long‐term culture of GFP and VGM groups. g) Summary of the CD34^+^CD41^+^ rate in the long‐term culture of GFP and VGM groups, calculated by flow cytometry. h) Graph showing normalized calculations based on data from figures h) and i), providing standardized reference points. Data are means ± SD of biologically independent samples. Statistical significance was calculated using a two‐tailed *t*‐test. The statistical significance indicated in the figures was assigned as not significant (ns) *p* > 0.05; ^*^ *p* < 0.05; ^**^ *p* < 0.01; ^***^ *p* < 0.001; ^****^ *p* < 0.0001.

MkPs are unipotent progenitors with limited proliferative capacity, making their expansion critical for large‐scale platelet production.^[^ ^4^ ^]^ To assess the proliferative potential of VGM‐induced MkPs, we performed EdU incorporation assays. The results showed that MkPs induced by VGM had a proliferation rate that was significantly higher than that of the control group (49.10% ± 5.51% vs 33.40% ± 0.86%) at day10 (**Figure** **2**a,b). Furthermore, the VGM group exhibited a significantly lower percentage of apoptotic cells (2.06% ± 0.40% vs 7.31% ± 0.40%), indicating enhanced cell survival (Figure 2c,d). Additionally, we evaluated the apoptosis rates of cells cultured long‐term until day 16 and found that the VGM group maintained a lower apoptosis rate compared to the GFP group (6.15% ± 0.32% vs 14.58% ± 0.47%) (Figure 2e). These results showed that VGM treatment continued to support better cell survival even in prolonged cultures.

To further investigate the effects of VGM treatment on cell expansion and survival, routine cell counting was conducted and confirmed an increased proliferation rate, with the total cell count in the VGM group being four times that of the control group by day 25 (Figure 2f). The VGM‐treated cells continued to expand and remained viable for at least 51 days (Figure 2f). During expansion, the VGM group maintained a higher proportion of CD34^+^CD41^+^ expression, though this proportion gradually declined in late passages of MkPs after extended culture (Figure 2g). The number of CD34^+^CD41^+^ MkPs in the VGM‐treated cells is also much higher than that in the control group during extended culture (Figure 2h).

These results demonstrate that VGM treatment significantly enhances the proliferative capacity of MkPs, facilitating both their proliferation and the maintenance of their progenitor state over an extended culture period.

### 2.3 Increased Yield of Mature MKs from VGM‐Derived MkPs

**Figure 3:**
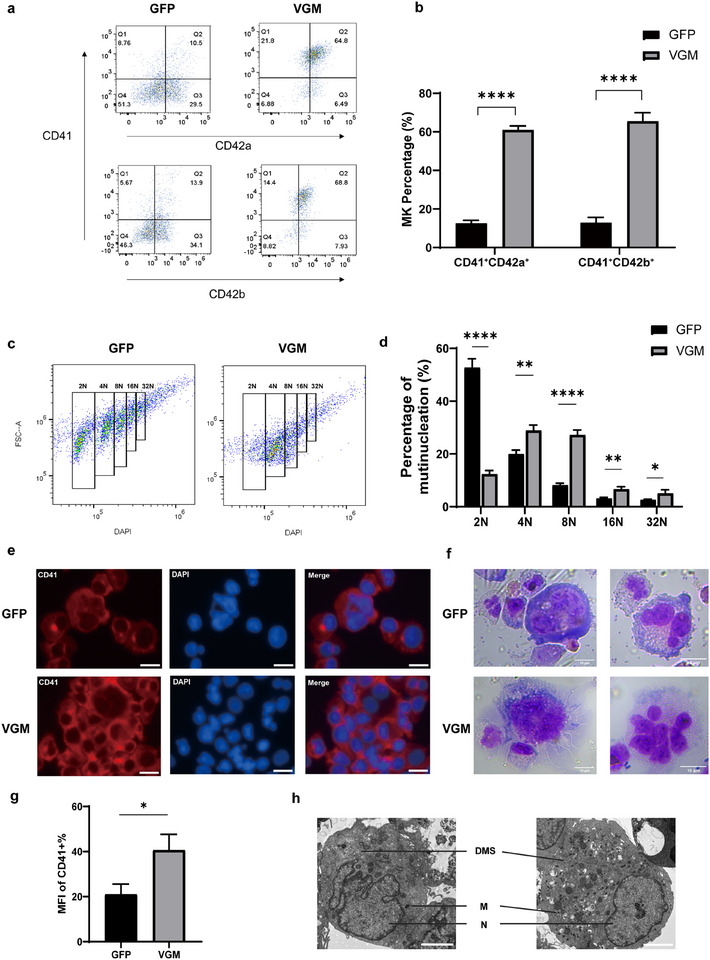
VGM cocktail improved the production of mature MKs. a) Representative dot plots from flow cytometric analysis showing the expression of CD41, CD42a, and CD42b on cells of GFP and VGM groups on differentiation day 17. b) Quantification of CD41^+^CD42a^+^ and CD41^+^CD42b^+^ cells in (a). c) Flow cytometry analysis of DNA ploidy on differentiation day 17. Multinucleation was categorized into 2N, 4N, 8N, 16N, and 32N. d) Statistical analysis of multinucleation (2N‐32N) in GFP and VGM groups on differentiation day 17. e) Representative images of cells stained with CD41 (red) and DAPI (blue) on differentiation day 17. Scale bar, 20 µm. f) MFI quantification of CD41 expression in (e). g) Representative image of Wright‐Giemsa staining in VGM groups on differentiation day 17. Scale bar, 10 µm. h) Electron microscopic images of Day 17‐MKs. The left, GFP‐MKs; the right, VGM‐MKs. DMS, demarcation membrane system; G, granule; N, nucleus. Scale bars represent 5 µm. Data are means ± SD of biologically independent samples. Statistical significance was calculated using a two‐tailed *t*‐test. The statistical significance indicated in the figures was assigned as ^*^ *p* < 0.05; ^**^ *p* < 0.01; ^****^ *p* < 0.0001.

To evaluate the maturation potential of VGM‐induced MkPs, cells were cultured for an additional 7 days in Stage 2 differentiation medium. The VGM group produced a significantly higher proportion of mature megakaryocytes (MKs) (CD41^+^CD42b^+^), ≈6 times that of the GFP group (72.90% ± 0.42% vs 12.15% ± 0.78%) (**Figure** **3**a,b). Additionally, 63.4% of these MKs co‐expressed CD41 and CD42a (compared to 10.79% ± 1.44% in the GFP group) (Figure 3b).

During maturation, MKs undergo endomitosis, resulting in highly polyploid cells with DNA content greater than 4N in a single polylobulated nucleus, essential for proplatelet formation.^[^ ^1c^ ^]^ As expected, VGM‐induced MKs exhibited polyploidization, with a maximum ploidy of 32N and a significantly higher proportion of 4N (28.93% ± 2.05% vs 20.03% ± 1.44% in the GFP group), 8N (27.2% ± 1.87% vs 8.16% ± 0.78%), 16N and 32N cells, indicating a greater propensity for mature, multinucleated states (Figure 3c,d). Immunofluorescence and Wright–Giemsa staining confirmed the presence of MKs with characteristic morphology, including extended long branches, increased cell size, and polyploidy, indicative of terminal maturation and imminent proplatelet formation (Figure 3e–g). Scanning electron microscope of VGM‐induced MKs revealed characteristic megakaryocyte organelles, such as the nucleus, granules, and the demarcation membrane system (DMS) (Figure 3h).

These findings underscore the VGM cocktail's ability to significantly enhance the in vitro production of mature MKs.

### 2.4 Efficient Platelet Production In Vitro and In Vivo from VGM‐Induced MkPs

**Figure 4:**
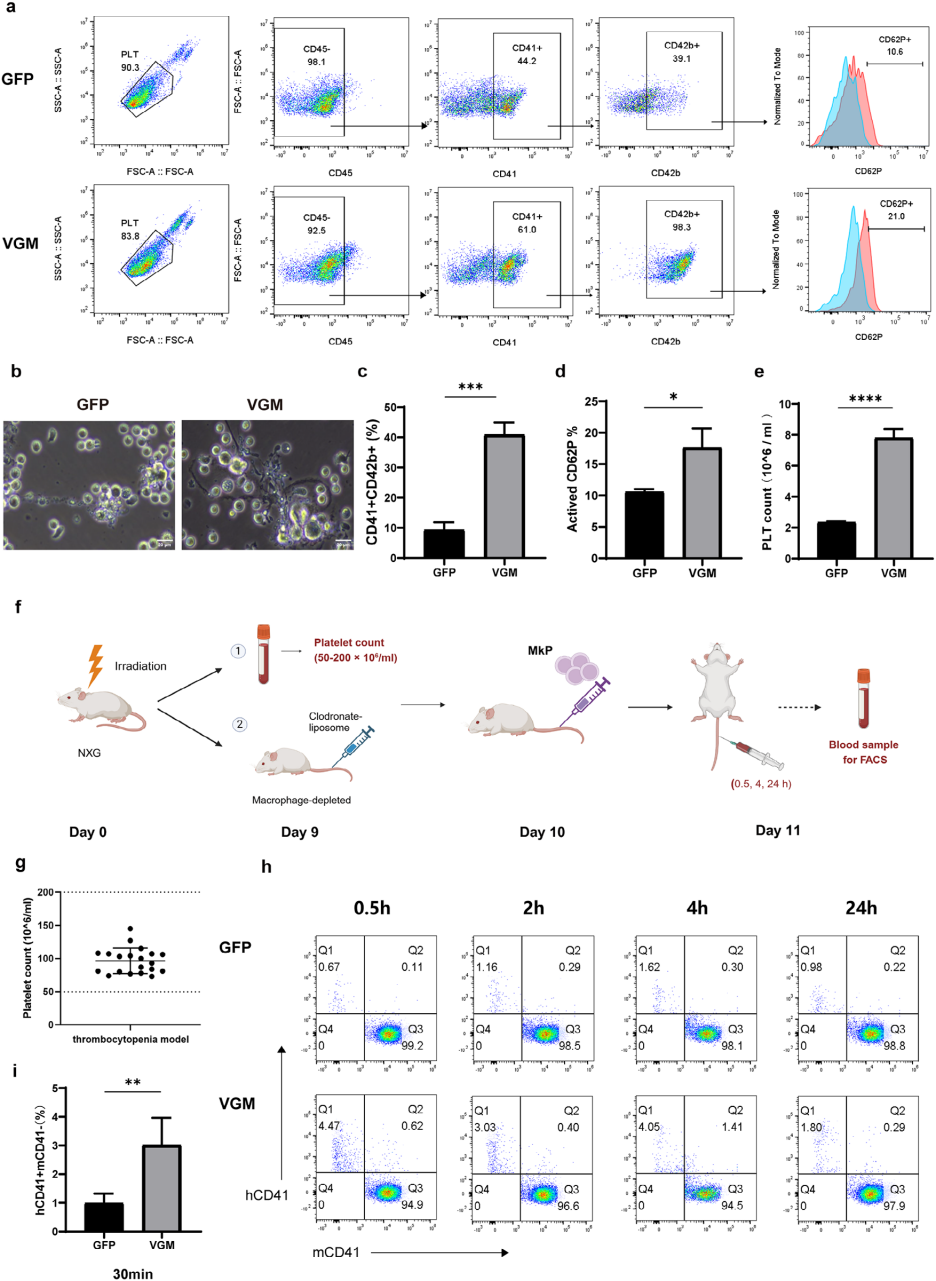
Efficient platelet production in vitro and in vivo from VGM‐induced MkPs. a) Flow cytometry analysis of platelet populations (CD45^−^CD41^+^CD42b^+^) on differentiation day 20 in GFP and VGM groups in vitro. Platelets were activated with ADP and TRAP‐6. Blue, unactivated platelets; red, activated platelets. b) Phase‐contrast microscopy images of filamentous platelets differentiated at day 20. Scale bar, 20 µm. c) Quantification of CD41^+^CD42b^+^ platelets in (a). d) Statistical analysis of CD62P^+^ in activated platelets (CD45^−^CD41^+^CD42b^+^) under different treatment conditions, as shown in (a). e) Absolute platelet counts on Day 10 of Stage two differentiation, as measured by flow cytometry with CD41. f) Schematic of the establishment of a thrombocytopenia mouse model and the in vivo differentiation of MkPs into platelets. g) Day 9 platelet count in the mouse model of thrombocytopenia (*n* = 15). h) Flow cytometry analysis of platelet differentiation from GFP and VGM MkPs in mice. CD41^+^ chimerism (mCD41^−^hCD41^+^) was measured at 0.5, 2, 4 and 24 h post injection. (i) CD41^+^ chimerism (mCD41^−^hCD41^+^) at 30 min post injection (*n* = 5). Data are means ± SD of biologically independent samples. Statistical significance was calculated using a two‐tailed *t*‐test and an ordinary one‐way ANOVA. The statistical significance indicated in the figures was assigned as ^*^ *p* < 0.05; ^**^ *p* < 0.01; ^***^ *p* < 0.001; ^****^ *p* <0.0001.

To assess platelet production, MKs were cultured for an additional 3 days, at 39 °C, after which proplatelet‐like morphology could be readily observed in the VGM group, exhibiting multiple protrusions with long branches (**Figure** **4**a,b). The VGM group produced significantly more CD41^+^CD42b^+^ platelets (40.9%±4.07%) compared to the control group (9.27% ± 2.54%) (Figure 4c). To evaluate the functionality of the generated platelets, we incubated the platelets on day 20 of differentiation with ADP and TRAP‐6. The results revealed a significantly higher percentage of CD62P expression in platelets from the VGM‐treated MkPs compared to those from the GFP group (17.63% vs 10.60%), indicating enhanced activation (Figure 4d). Importantly, their activation patterns closely resembled those of human peripheral blood‐derived platelets (Figure S3a, Supporting Information). By day 10 of Stage 2 differentiation, based on absolute platelet counts, we deduce that one VGM‐D10 cell can produce ≈7.81 platelets, whereas the control cells can yield ≈2.43 platelets (Figure 4e).

The capacity of VGM‐induced MkPs to mature and release platelets in vivo was evaluated using a thrombocytopenia model established in NXG mice via irradiation as described previously^[^ ^19^ ^]^ (Figure 4f), where mouse platelet counts ranged from 50 to 200 × 10^6^ mL^−1^ by day 9 (Figure 4g). After macrophage depletion, MkPs derived from VGM and GFP treatments were intravenously transfused into the mice. The release of human platelets in the mouse peripheral blood was assessed at 0.5, 2, 4, and 24 h post injection.

Notably, human CD41^+^ platelet chimerism (mCD41^−^hCD41^+^) in the VGM group was successfully detected and reached variable levels: 4.47% at 0.5h , 3.03% at 2 h, 4.05% at 4 h, and 1.80% at 24 h post‐injection, all of which are much higher than those in the control group (Figure 4h,i). GFP‐expressing nucleated cells and platelets were detected, allowing tracking of MkP differentiation into MKs and subsequent platelet production in situ (Figure S3b–d, Supporting Information). Based on chimerism data, it was estimated that each VGM‐induced MkP differentiated into ≈8 platelets in vivo, underscoring the differentiation and tracking potential of these MkPs. However, this likely significantly underestimates the true capacity due to MK cell death post‐injection and platelet rejection by residual innate immune cells. This result validated the potential of VGM‐induced MkPs to differentiate into platelets in vivo and demonstrated that long‐term culture in vitro didn't affect their differentiation capacity.

Collectively, the VGM cocktail‐derived MkPs possess considerable potential for differentiation into platelets both in vitro and in vivo.

### 2.5 VGM‐Induced MkPs Exhibit Distinct Gene Expression Signatures

**Figure 5:**
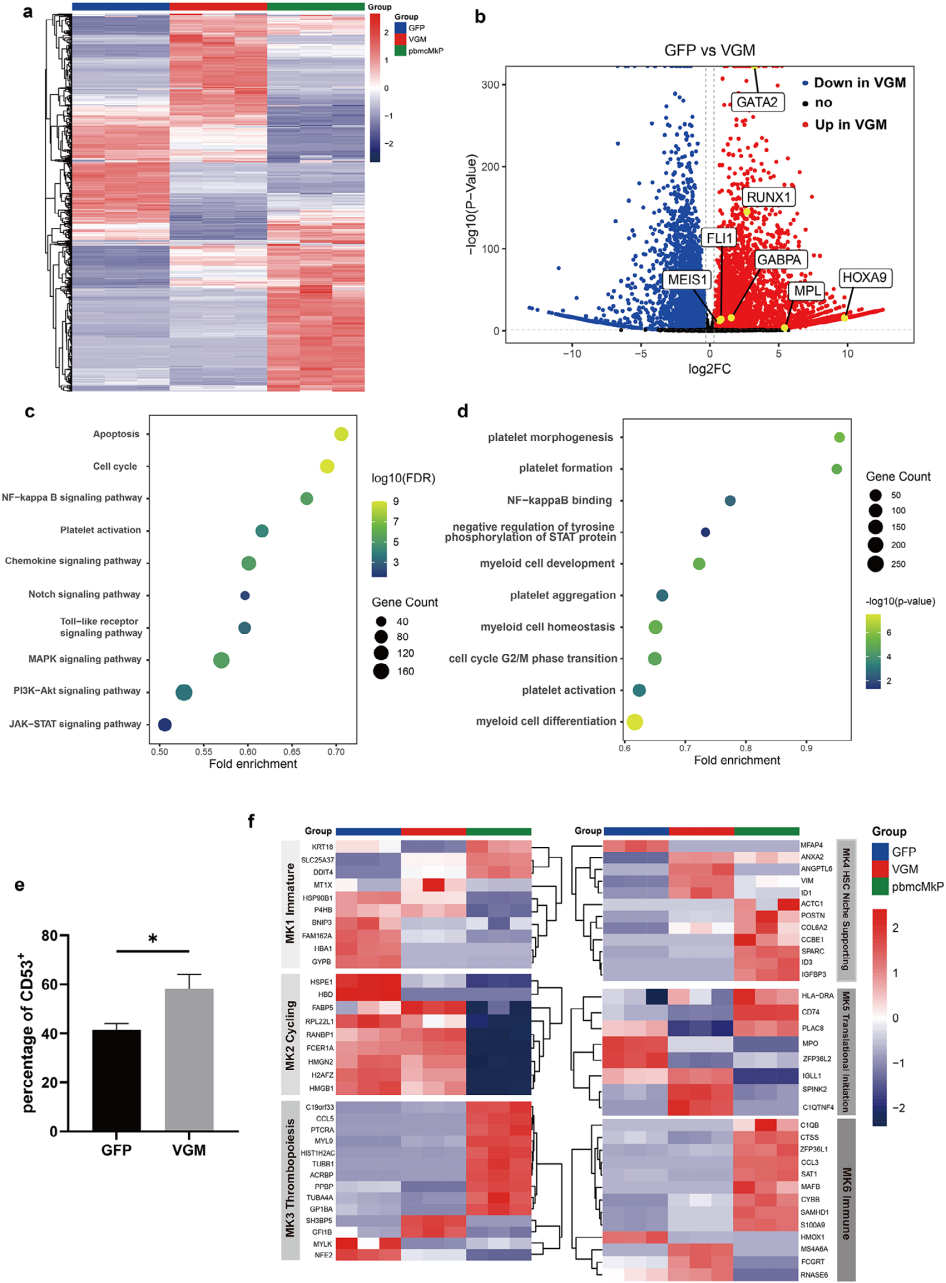
Significant transcriptional response induced by VGM cocktail. a) Heatmap analysis of MkPs derived from GFP (*n* = 3), VGM (*n* = 3) and PBMC (*n* = 3) groups. Genes are clustered based on their expression profiles across the three groups. b) Volcano plot displaying significantly upregulated (red) and downregulated (blue) genes in VGM‐derived MkPs compared to GFP‐derived MkPs. The *x*‐axis represents the log2 fold change, and the *y*‐axis represents the ‐log10 *p*‐value. Red or blue dots indicate DEGs identified by *p*‐value < 0.05 and |log2(FoldChange)|>0.3. Interested genes were marked as yellow dots. c) KEGG pathway analysis of MkPs from the VGM group compared to those from the GFP group. d) GO enrichment analysis of MkPs from the VGM group compared to those from the GFP group. e) TPM analysis of the CD53 expression on MkPs from GFP and VGM groups at day 10. f) Heatmap analysis of MkPs of the GFP (*n* = 3), VGM (*n* = 3) and PBMC (*n* = 3) groups, based on MK1‐MK6 gene sets. Data are means ± SD of biologically independent samples. Statistical significance was calculated using a two‐tailed *t*‐test. The statistical significance indicated in the figures was assigned as not significant (ns) *p* > 0.05; ^****^ *p* < 0.0001.

To elucidate the molecular mechanisms underlying VGM treatment, we performed RNA‐seq analysis on MkPs from the GFP and VGM‐treated groups after differentiation for 10 days, along with MkPs isolated from human PBMCs for comparison (Figure S4a,b, Supporting Information). The heatmap in **Figure** **5**a illustrates distinct gene expression patterns in the VGM and GFP groups compared to PBMC‐derived MkPs, highlighting significant regulatory differences. Several MkP‐related genes, including *FLI1*, *GATA2*, *RUNX1*, and *MPL*, were significantly upregulated in the VGM‐treated group (Figure 5b). Kyoto Encyclopedia of Genes and Genomes (KEGG) pathway analysis identified significant enrichment in NF‐kappa B, MAPK, cGMP‐PKG, and PI3K‐AKT signaling pathways in VGM‐induced MkPs, all previously associated with MkP differentiation and platelet development (Figure 5c).^[^ ^20^ ^]^ Gene Ontology (GO) analysis further revealed significant enrichment in gene sets related to myeloid cell development, platelet formation, and cell cycle regulation in the VGM group, consistent with the robust production of CD34^+^CD41^+^ MkPs and their enhanced proliferative capacity (Figure 5d).

Emerging evidence suggests that MKs are heterogeneous, comprising at least three subpopulations: platelet‐producing MKs, immune‐related MKs, and niche‐supporting MKs.^[^ ^21^ ^]^ Among these, platelet‐producing MKs are specialized for platelet generation. In MKs induced via direct chemical reprogramming from erythroblasts, a subset expresses CD53, a surface marker indicative of immune‐related MKs.^[^ ^22^ ^]^ These studies hint that MkPs may also consist of different subtypes. We then compared the expression of *CD53* and found that MkPs from the VGM and GFP groups exhibited significantly lower expression of *CD53* at the transcriptional level compared to those from PBMCs (Figure 5e), suggesting that our differentiation system is optimized for generating thrombopoiesis‐biased MkPs. Consistent with this, when mapping the gene expression profiles of VGM‐treated and control MkPs onto the six subtypes identified by Wang et al.^[^ ^20^, ^23^ ^]^ (Figure 5f), we found reduced expression of immune‐related programs (enriched in MK6) in both differentiated MkP groups compared to PBMC‐derived MkPs. Besides, VGM‐induced MkPs showed notable characteristics akin to niche‐supporting MKs, similar to the MK4 subtype, when compared to GFP‐treated MkPs.

Collectively, these transcriptomic analyses indicate that VGM‐treated MkPs are predominantly committed to platelet production but retain heterogeneity with characteristics of other MK subpopulations.

### 2.6 JAK2/STAT3 Signaling Pathway Mediates VGM‐Induced MK Lineage Commitment

**Figure 6:**
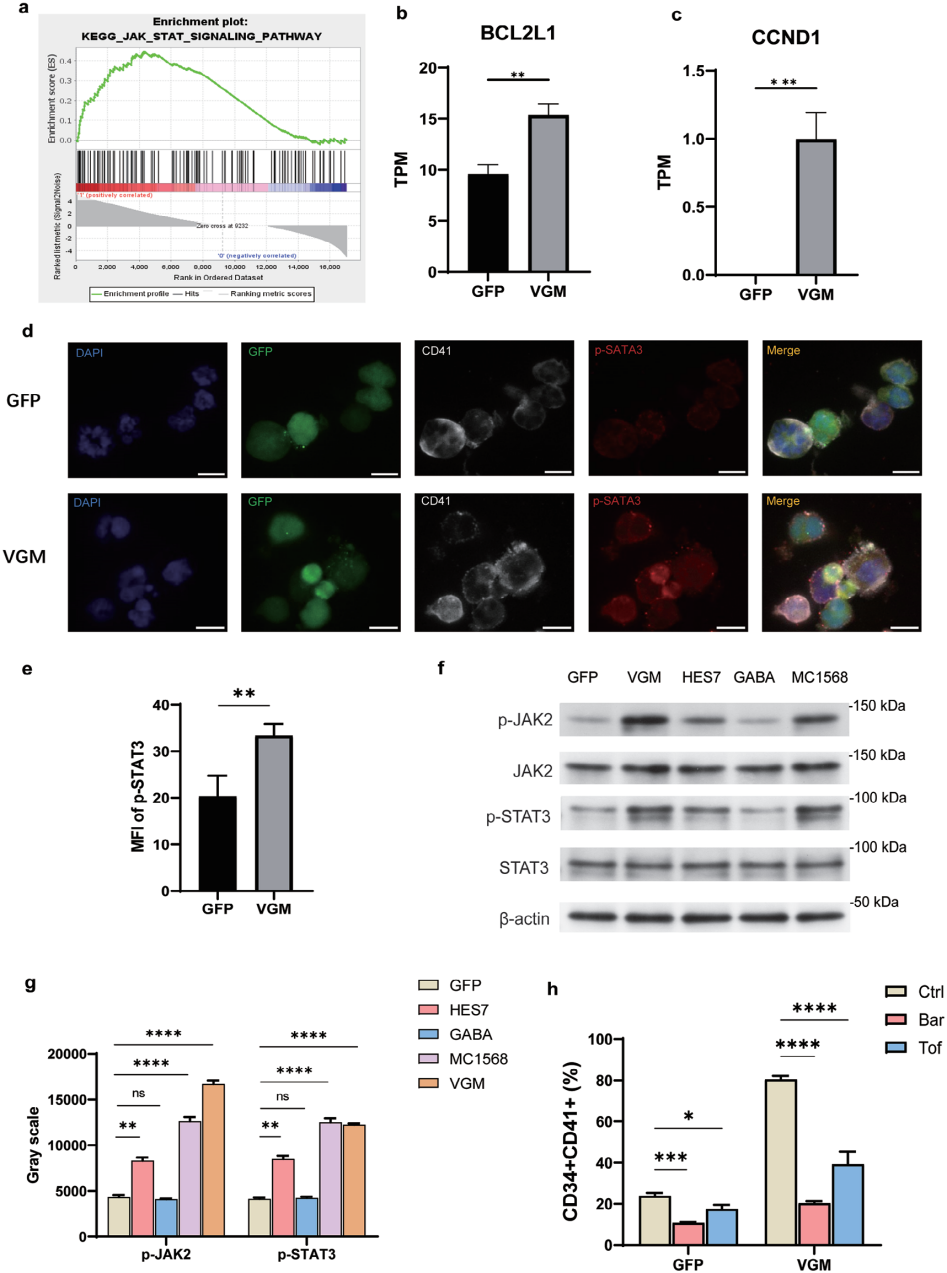
VGM cocktail promoted MkP production via the JAK2/STAT3 signaling pathway. a) GSEA analysis of the JAK/STAT signaling pathway in MkPs from the VGM group compared to those from the GFP group. b,c) TPM analysis of BCL2L1 b) and CCND1 c) in GFP and VGM groups. d) Immunofluorescence images of cells on differentiation day 17, displaying GFP fluorescence (green), along with staining for DAPI (blue), CD41 (gray), and p‐STAT3 (red). Scale bar, 10 µm. e) MFI quantification of p‐STAT3 in (d). f) Western blot analysis of JAK2, p‐JAK2, STAT3, and p‐STAT3 in MkP cells derived from GFP, VGM, HES7, GABA, MC1568‐treated groups on differentiation day 10. g) Grayscale intensity analysis of p‐JAK2 and p‐STAT3 bands from (f) in MkP cells derived from different groups. h) Flow cytometry analysis of MkP cells (CD34^+^CD41^+^) of GFP and VGM groups on differentiation day 10, treated with JAK2 pathway inhibitors Baricitinib (Bar) and Tofacitinib (Tof). Data are means ± SD of biologically independent samples. Statistical significance was calculated using a two‐tailed *t*‐test and an ordinary one‐way ANOVA. The statistical significance indicated in the figures was assigned as not significant (ns) *p* > 0.05; ^*^ *p* < 0.05; ^**^ *p* < 0.01; ^***^ *p* < 0.001; ^****^ *p* < 0.0001.

Previous studies have highlighted the pivotal role of the JAK2‐STAT3/5 signaling pathway in TPO/c‐MPL‐mediated megakaryocyte differentiation. TPO activates JAK2, which subsequently phosphorylates STAT family members, leading to the nuclear translocation of activated STAT3 and regulation of downstream gene expression.^[^ ^1c^ ^]^ Consistent with these findings, our KEGG and GO analyses demonstrated significant enrichment of JAK/STAT‐related gene sets (KEGG: hsa04630 and GO:0042532) in the VGM group (Figure 5c,d). Further validation using Gene Set Enrichment Analysis (GSEA) confirmed the upregulation of the JAK‐STAT signaling pathway gene set (**Figure** **6**a). Moreover, the expression of *CCND1* and *BCL2L1*, genes regulated by phosphorylated STAT3 (p‐STAT3), was markedly upregulated in VGM‐treated MkPs (Figure 6b,c). These two genes are involved in cell cycle progression and anti‐apoptotic processes, respectively, suggesting that VGM treatment promotes MK lineage commitment, at least in part, through JAK2/STAT3 signaling.

**Figure 7:**
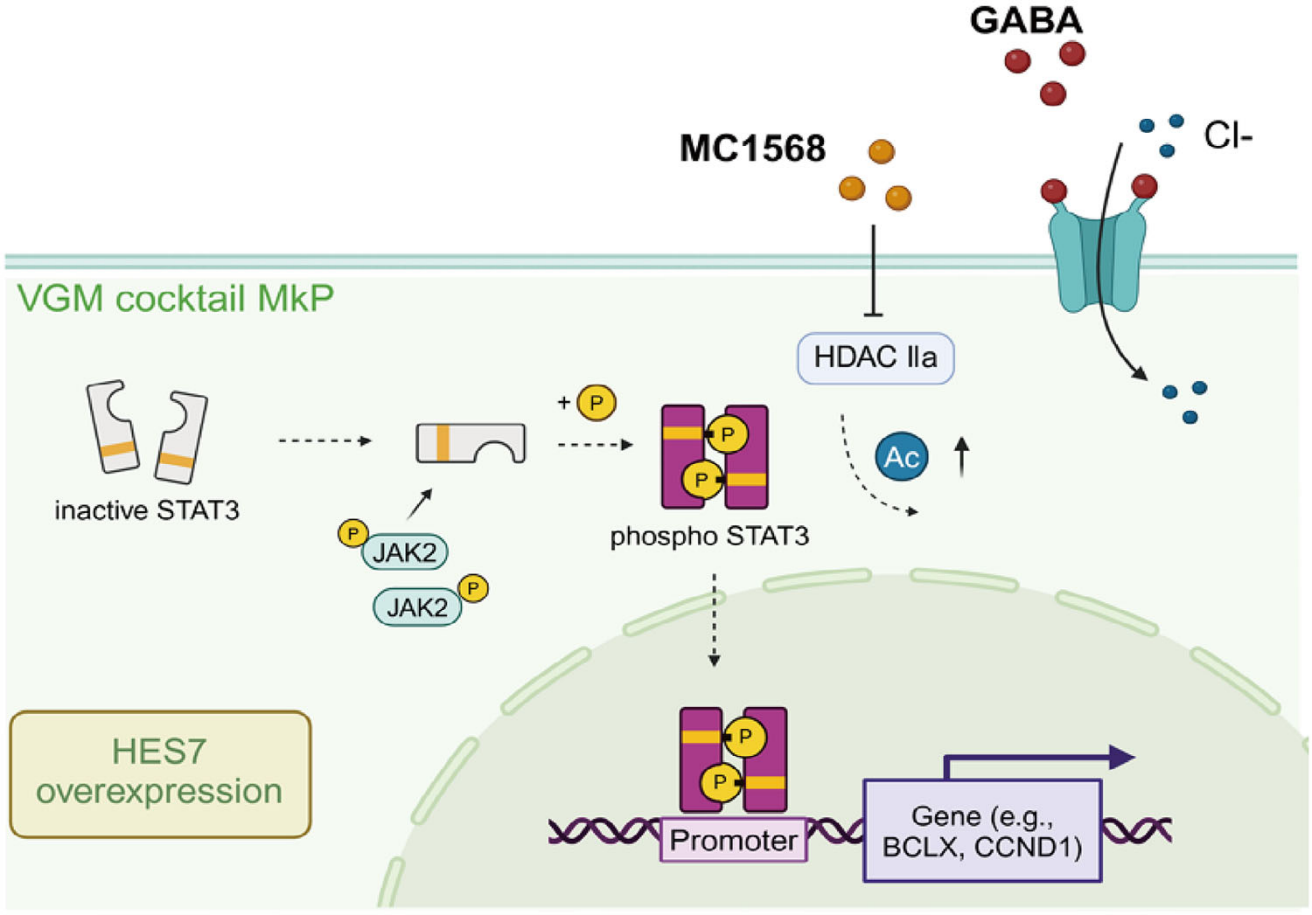
Mechanistic diagram illustrating the pathways and mechanisms involved in the enhanced differentiation of MkP induced by VGM cocktail.

To validate this hypothesis, we assessed the activation status of the JAK2/STAT3 pathway in 10‐day differentiated MkPs. Immunofluorescence staining revealed a significant increase in p‐STAT3 levels in VGM‐induced MkPs compared to the GFP group (33.38% ± 2.53% vs 20.27% ± 4.50%) (Figure 6d,e), which was corroborated by Western blot analysis (Figure 6f,g). As expected, the effect of the VGM cocktail on MK lineage commitment was substantially diminished when JAK2 inhibitors, specifically Baricitinib^[^ ^24^ ^]^ and Tofacitinib,^[^ ^11^ ^]^ were introduced, leading to a ≈60% and ≈40% reduction in the CD34^+^CD41^+^ MkP population, respectively (Figure 6h). To determine which of the three independent factors (HES7 overexpression, HDAC inhibitor, and GABA agonist) primarily contributes to the activation of the JAK2/STAT3 signaling pathway, we conducted Western blot analyses on cells treated separately with these factors. The results showed that HES7 and MC1568 enhanced the phosphorylation levels of JAK2 and STAT3, while VGM treatment induced significantly higher p‐JAK levels than all other treatments (Figure 6f,g). These findings strongly suggest that the VGM cocktail promotes MkP production via the JAK2/STAT3 signaling pathway (**Figure** **7**).

## 3 Discussion

Our study introduces a novel approach for the production of MkPs with high efficiency, which reaches as high as 90%. This approach integrates the overexpression of HES7, the use of GABA, and the HDAC inhibitor MC1568, collectively referred to as the VGM cocktail. The MkPs generated not only maintain their proliferative capacity for ≈4 weeks in vitro but also demonstrate the ability to mature into MKs under differentiation conditions. Notably, a substantial number of platelets were produced both in vitro and in vivo. Transcriptomic analysis further revealed that the JAK2/STAT3 signaling pathway plays a central role in mediating the VGM cocktail's effects on MK lineage commitment.

Previous research has shown that TPO/cMPL‐mediated JAK2/STAT3 signaling activation promotes the differentiation of HSPCs into the MK lineage.^[^ ^9^, ^15^ ^]^ Our study extends this understanding by demonstrating that the JAK2/STAT3 pathway is also required for the enhanced MK lineage differentiation observed with the VGM cocktail. Specifically, the Notch effectors HES1/5 have been shown to increase the efficiency of STAT3 phosphorylation.^[^ ^8b^ ^]^ In our study, HES7, which belongs to the HES gene family and interacts with Notch signaling, similarly promotes STAT3 activation. Moreover, HDAC7 has been reported to suppress STAT3 activation through deacetylation. Consistent with this, MC1568, which inhibits HDAC class IIa, is shown to enhance STAT3 phosphorylation within our cocktail.^[^ ^9^ ^]^ Although GABA treatment alone fails to induce JAK2 and STAT3 activation, cells from the VGM cocktail treatment group express the highest level of phosphorylated JAK2 and STAT3, indicating a synergistic effect among these factors.

Despite the significant enhancement in MkP differentiation efficiency achieved with our approach, several limitations remain. We observe a decline in the proportion of MkPs with extended culture (Figure 2f–i). The decrease in MkPs during extended cultures might be influenced by factors like cellular senescence or metabolic waste accumulation, rather than nutrient depletion from our long‐term culture medium. Future experiments aimed at addressing these issues could help maintain MkP numbers and improve the efficiency of producing megakaryocytes and platelets. Understanding these dynamics could enhance our differentiation protocols and culture conditions.

In this study, we explored the phenotypic and gene expression characteristics of MkPs treated with VGM and the control group. Our data indicate that MkPs treated with VGM exhibit notable heterogeneity in gene expression, suggesting they are at a relatively early stage of differentiation. This expression diversity, similar to that reported in more mature cells, implies that these MkPs might differentiate into various cell subtypes in the future, even under high induction conditions.^[^ ^6^ ^]^ Particularly, we observed a higher proportion of genes associated with the early megakaryocyte subtypes MK1 and MK2 in the VGM‐treated group. This finding suggests that while these MkPs are less mature, they have already begun to demonstrate the potential to differentiate into diverse MK subtypes. This early stage of differentiation provides a crucial foundation for further functional studies and optimization of platelet production.^[^ ^14^ ^]^ Our research underscores the need to refine our induction methods to more precisely control MkP differentiation into platelet‐producing subtypes, enhancing platelet yield and functionality.

In conclusion, our findings demonstrate that the VGM cocktail significantly enhances the generation of MkPs, leading to increased production of mature MKs and platelets from human HSPCs. The JAK2/STAT3 signaling pathway is a pivotal mediator of the VGM cocktail's effects on MK lineage commitment. The VGM cocktail offers a promising strategy for large‐scale in vitro production of MKs and platelets, laying the groundwork for advancements in platelet transfusion therapies and enriching our understanding of megakaryocyte differentiation to foster the development of new treatment strategies.

## 4 Experimental Section

### Plasmids

The pCDH‐MSCV‐hCD38‐EF1α+Puro cloning and expression vector (Plasmid #134936) was purchased from Addgene. cDNAs of candidate genes were synthesized by GeneCreate Biological Engineering Co., Ltd. and inserted under the MSCV promoter. The vector containing an inserted non‐functional sequence was used as the control.

### Differentiation of HSPCs

Peripheral blood CD34^+^ cells were purchased from Shanghai MiaoShun Biotechnology Co., Ltd. The cells were cultured in SFEMII (09655, StemCell Technologies), supplemented with 20 ng mL^−1^ each of hIL‐3 (200‐03, PEPROTECH), hIL‐6 (200‐06‐100, PEPROTECH), TPO (300‐18‐100, PEPROTECH), hSCF (300‐07‐100, PEPROTECH), and Flt3 Ligand (300‐19‐100, PEPROTECH), at 37 °C in 5% CO_2_ for 10 days, or until collection for analysis. Chemical compounds, including Mocetinostat (18287, Cayman), MC1568 (16265, Cayman), Quisinostat (14088, Cayman), GABA (0344, Tocis), Baricitinib phosphate (5.7 nm; HY‐15315A, MCE) and Tofacitinib (20 nm; HY‐40354, MCE), were added to the culture medium during this stage. Half of the culture medium was replaced every day.

In Stage 2, the cells were cultured in IMDM containing N2, B27 (17504044, Gibco), 20 ng mL^−1^ each of TPO, hSCF, and hIL‐6 at 39 °C in 5% CO_2_ for 7–10 days. Half of the culture medium was replaced every day.

### Virus Production

The 293T cells were cultured in high‐glucose DMEM containing 10% FBS, 100 µm nonessential amino acids, 100 µm glutamine, and 100 U mL^−1^ penicillin‐ streptomycin (15140‐122, Gibco). Virus production was conducted using the calcium phosphate transfection method. The cDNA vectors, packaging vector psPAX2 (Plasmid #12260), and envelop vector pMD2.G (Plasmid #12259) were mixed at a 3:2:1 ratio. The viral supernatant was collected at 48 and 72 h post‐transfection, filtered via a 0.45 µm filtration unit, and concentrated using the 100 kDa Column (Millipore) to different final volumes.

### Virus Transduction

RetroNectin Recombinant Human Fibronectin Fragment (T100A, Takara) was used to pre‐coat the plates at a concentration of 4–20 µg cm^−^^2^ at 4 °C overnight. Following the coating, plates were blocked with 2% BSA in PBS for 30 min and subsequently washed with PBS. Viral suspension was added, and a secondary infection was conducted 24 h later. The selection was initiated using 1 µg mL^−1^ puromycin (HY‐B1743A, MCE).

### EdU Staining

Cells were plated in 96‐well plates and incubated with 10 µm EdU (Lumiprobe) for 4 h at 37 °C in 5% CO_2_. After the EdU incubation, the cells were centrifuged to remove the supernatant and washed once with PBS. The cells were then transferred onto glass slides using a pipette, allowed to air‐dry, and subsequently fixed with 4% (wt/vol) formaldehyde (Sigma‐Aldrich). Cells were rinsed with 100 mM Tris (Shanghai Yuanye Bio‐Technology), to remove any excess formaldehyde. For staining, cells were first permeabilized with 0.1% Triton X‐100 (Beyotime, China), then treated with freshly prepared EdU staining solution for 30 min in a dark environment at room temperature. This was followed by staining with 2 µg mL^−1^ DAPI (Cell Signaling Technology) for 20 min in the dark at RT. The slides were mounted and left to dry overnight. Imaging was conducted using a Leica DM6 B microscope, and ImageJ software was used for image analysis.

### Flow Cytometry Analysis and Sorting

The following cell surface markers were used for flow cytometry analysis:

- MkP: CD34 and CD41.
- MK and PLT: CD41, CD42a, and CD42b.

The antibodies used in this study are APC‐Cy7 CD34, APC CD41, PE CD42a, and PE CD42b, along with live/dead dyes PI and DAPI (BioLegend).

Flow cytometry was performed on BD FACSAria II (BD Biosciences). Flow cytometry sorting was performed on FACS Aria (BD Biosciences) and the data was analyzed with FlowJo (Tree Star).

### Colony‐Forming Unit‐MK Assay

The colony‐forming unit assay was conducted using the MegaCult‐C Complete Kit with Cytokines (04971, Stemcell Tech), following the manufacturer's instructions, with starting cells being those infected and drug‐selected on Day 5 of differentiation. After 12–14 days of culture, colonies were counted, and megakaryopoietic colonies were stained using the antibody provided in the kit.

### Flow Cytometry Sorting of MkP Cells

MkPs were obtained from either human PBMC or 10 days of Stage 1 differentiation of CD34^+^ cells isolated from cord blood. MkPs were isolated by flow cytometry using a multi‐parameter staining approach with the following antibodies: AmCyan Lineage Cocktail (Lin), PE‐Cy7 CD34, PE‐Cy5 CD38, APC CD123, AF700 CD45RA, and PerCP‐Cy5.5 CD41. MkP cells were defined as Lin^−^CD34^+^CD38^+^CD123^−^CD45RA^−^CD41^+^.

The gating strategy for identifying MkP cells involved the following steps: Lin^−^ cells were gated to exclude mature lineage‐positive cells, followed by selecting CD34^+^CD38^+^ cells from the Lin^−^ population. CD123^−^CD45RA^−^ cells were then identified within the CD34^+^CD38^+^ population. Finally, CD41^+^ cells were gated to identify the MkP population. The sorted MkP cells were then collected for further downstream analysis, including transcriptomic profiling and differentiation assays.

### RNA‐Sequencing

MkP cells were used to generate Smart‐Seq2 libraries at Beijing GeekGene Technology Co., Ltd, and subject to RNA‐sequencing analysis. DEG, KEGG, and GO analysis were performed with the Geekgene Bioinformatics Service Portal/Platform (http://bioinfo.geekgene.com.cn/). Heatmaps were generated in R (4.4.0) using the pheatmap package (1.0.12). Volcano plots were made in R (4.4.0) using the ggplot2 package (3.5.1). GSEA analysis was performed in GSEA software (4.3.2).

### Immunofluorescence Staining Assay

For immunofluorescence staining, cells were fixed with 4% paraformaldehyde for 15 min at room temperature, followed by permeabilization with 0.1% Triton X‐100 for 5 min. After blocking with 1% BSA in PBS for 30 min, the cells were incubated with primary antibodies (1:200 dilution) overnight at 4 °C, followed by incubation with secondary antibodies for 1 hour at room temperature. After three washes with PBS, the cells were mounted using ProLong Gold Antifade Mountant with DAPI (Invitrogen). Images were acquired using LEICA TCS SP5.

The antibodies used for immunofluorescence staining are: anti‐CD41 (Abcam, ab181582), anti‐p‐STAT3 (CST, 4113S), Alexa Fluor 594 Goat Anti‐Mouse IgG (Abcam, ab150116), and Alexa Fluor 647 Goat anti‐Rabbit IgG (Invitrogen, A78957).

### Polyploidy Analysis

Cells were collected and pre‐stained with Alexa Fluor 647‐conjugated anti‐CD41 antibody (BioLegend, 133914), followed by fixation with 70% cold ethanol and incubated overnight at 4 °C. Cells were then stained with DAPI (Sigma‐Aldrich, D9542) in 0.1% Triton X‐100 and analyzed using Beckman Coulter CytoFLEX S. Data was analyzed with FlowJo.

### Electron Microscopy

Electron microscopy was done with GFP‐ and VGM‐treated differentiated MKs on day 17. Cell pellets were collected by centrifugation, and were fixed with electron microscopy fixative (Servicebio, G1102) at room temperature in the dark for 30 min. After fixation, samples were postfixed with 1% osmium tetroxide, dehydrated, infiltrated with epoxy resin, and embedded. Ultrathin sections (60–80 nm) were cut, stained with 2% uranyl acetate in 70% methanol and Reynolds’ lead citrate, and observed with a transmission electron microscope (HT‐7800; Hitachi, Tokyo, Japan). Imaging was performed by Sevicebio., Wuhan, China.

### Platelet Activation Assay

Platelets were purified from human peripheral blood and from cells differentiated in vitro. For activation, platelets were incubated with ADP (0.2 mm) and TRAP‐6 (0.05 mm) for 10 min. Following activation, platelets were stained with BioLegend antibodies: CD45 (APC‐CY7, 368516), CD41 (Pacific Blue, 303714), CD42b (PE, 303906), and CD62P (APC, 304910) for 30 min. Flow cytometry was performed to assess the expression of CD62P on CD45^−^CD41^+^CD42b^+^ gated platelets, with a control group of unactivated platelets (without ADP and TRAP‐6). Data was analyzed using FlowJo.

### Platelet Counting

Cell culture supernatant at Day 20 was added to BD Trucount Tubes (BD, 24134) preloaded with a lyophilized pellet of fluorescent beadsm, and stained with anti‐human PE‐CD41 (Biolegend, 984508) antibody. The absolute number of platelets was determined via flow cytometry by comparing PE‐CD41 positive events to bead events, with calculations performed using the software BD CellQuest Pro.

### In Vivo Experiments

#### Animal Grouping and Treatment

NXG male mice (11–13 weeks old at the time of irradiation) were used to evaluate the platelet production ability of MkP cells. All mice were acquired from the Institute of Zoology, Chinese Academy of Sciences. The mice were divided into the following groups:

- GFP: Injected with 1 × 10^6 MkPs cultured for 10 days in vitro (*n* = 5).
- VGM: Injected with 1 × 10^6 MkPs cultured for 10 days in vitro (*n* = 5).
- Control Group (NC): Injected with an equivalent volume of PBS (*n* = 5).

### In Vivo Experiments—Irradiation and MkP Infusion

Day 0: 24 NXG mice were irradiated with 1.8 Gy X‐rays to induce thrombocytopenia. Post‐irradiation care included housing in SPF barrier conditions, antibiotic‐treated drinking water (5% enrofloxacin solution, 1‰ dilution; Zhonglongshenli, Hefei), daily sunflower seed supplementation, and nesting materials.

Day 9: Complete blood count (CBC) and macrophage depletion. Blood samples were collected from the retro‐orbital sinus for CBC. Macrophages were depleted using LIPOSOMA liposome macrophage depletion reagent (0.5 mg in 100 µL per mouse; YS Biological Technology, 40337ES08). Mice with platelet counts between 50 and 200 × 10^6 mL^−1^ were selected for further experiments.

Day 10: Tail vein infusion of MkPs or PBS (200 µL per mouse) was performed ≈24 h after macrophage depletion.

### Reconstitution Assessment

Peripheral blood samples were collected via tail vein at 0.5, 2, 4, and 24 h postinfusion to assess donor cell chimerism (hCD41^+^ platelets) using flow cytometry. Anticoagulated blood (40 µL in 1:9 sodium citrate solution; Hefei Zhonglongshenli) was analyzed for donor cell chimerism. During flow cytometry analysis, hCD41^+^ and mCD41^+^ cells were identified using APC anti‐mouse CD41 (133914, BioLegend) and PE anti‐human CD41 (303706, BioLegend).

The number of platelets produced per injected MkP was calculated as follows:

(1) $$\mathrm{Platelets}/\mathrm{MkP}=\frac{\mathrm{Post}-\mathrm{modeling}\;\mathrm{platelet}\;\mathrm{concentration}\times \mathrm{Total}\;\mathrm{mouse}\;\mathrm{blood}\;\mathrm{volume}\times \mathrm{Average}\;\mathrm{hCD}\;41\;\mathrm{chimerism}\;\mathrm{rate}}{\mathrm{Number}\;\mathrm{of}\;\mathrm{injected}\;\mathrm{MkPs}}$$

### Real‐Time PCR Analysis

Total RNA was extracted from cells using RNAiso Plus (Takara, Japan), and then reverse transcribed using PrimeScript RT reagent Kit (Perfect Real Time, Takara) following the manufacturer's instructions. Real‐time PCR was performed using TB Green Premix Ex Taq GC (Perfect Real Time, Takara) on an ABI 7900HT Fast Real‐Time PCR System. Real‐time PCR results were analyzed using Expression Suite v1.1 and relative expression was calculated using the 2^‐ΔΔCT method.

### Protein Phosphorylation Analysis

Cells were lysed using RIPA buffer (Beyotime, P0013B) supplemented with a protease and phosphatase inhibitor cocktail (Beyotime, P1045). Protein concentrations were quantified using the BCA Protein Assay Kit (Beyotime, P0010). Proteins were separated by SDS‐PAGE and transferred to PVDF membranes using a Bio‐Rad electrophoresis system. The membranes were then probed with primary antibodies at the following dilutions: JAK2 (CST, 3230T) at 1:1500, STAT3 (CST, 30835S) at 1:1500, phospho‐JAK2 (CST, 3776S) at 1:1000, phospho‐STAT3 (CST, 30835S) at 1:2000, and β‐actin (CST, 8457T) at 1:3000. Blots were visualized using an Odyssey XF imaging system (LI‐COR, Nebraska, USA), and band quantification was performed using LI‐COR Image Studio software.

### Statistics

Experimental groups were compared using GraphPad Prism Software. Student's *t*‐test was used for paired comparison and on‐way ANOVA for multiple comparisons. The statistical significance indicated in the figures was assigned as not significant (ns) *p* > 0.05; ^*^ *p* < 0.05; ^**^ *p* < 0.01; ^***^ *p* < 0.001; ^****^ *p* < 0.0001.

### Ethics Approval Statement

All animal experimental procedures complied with ethical guidelines and received approval from the Institutional Animal Care and Use Committee of Shanghai Jiao Tong University (IACUC Approval Number: 202101082). All human blood samples were obtained from Zhoushan Putuo District People's Hospital, with ethical approval from their Ethics Committee (Ethical Approval Number: 2020KY04).

## Conflict of Interest

The authors declare no conflict of interest.

## Author Contributions

F.Z. designed the project and conceptualized the manuscript. H.L., L.W., and J.L. conducted experiments and contributed to manuscript writing. H.Y., Y.Q., and K.Z. supported experimental procedures.

## Supporting information



Supporting Information

## Data Availability

The data that support the findings of this study are available from the corresponding author upon reasonable request.
