# Supplementary material for: Efficient Generation of Megakaryocyte Progenitors and Platelets From HSPCs via JAK2/STAT3 Signaling
Source: Adv Sci (Weinh). 2025 Apr 29;12(23):2500612. doi: 10.1002/advs.202500612 (PMC12199311; doi:10.1002/advs.202500612)
Supplement: Supplementary file 1 — Supporting Information [file ADVS-12-2500612-s001.docx]

Efficient Generation of Megakaryocyte Progenitors and Platelets from HSPCs via JAK2/STAT3 Signaling

Huicong Liu^1†^, Lingna Wang^1†^, Jiaqing Liu^1†^, Haitao Yuan^1^, Kaiqing Zhang^1^, Yun Qiu^1^, Fangfang Zhu^1*^


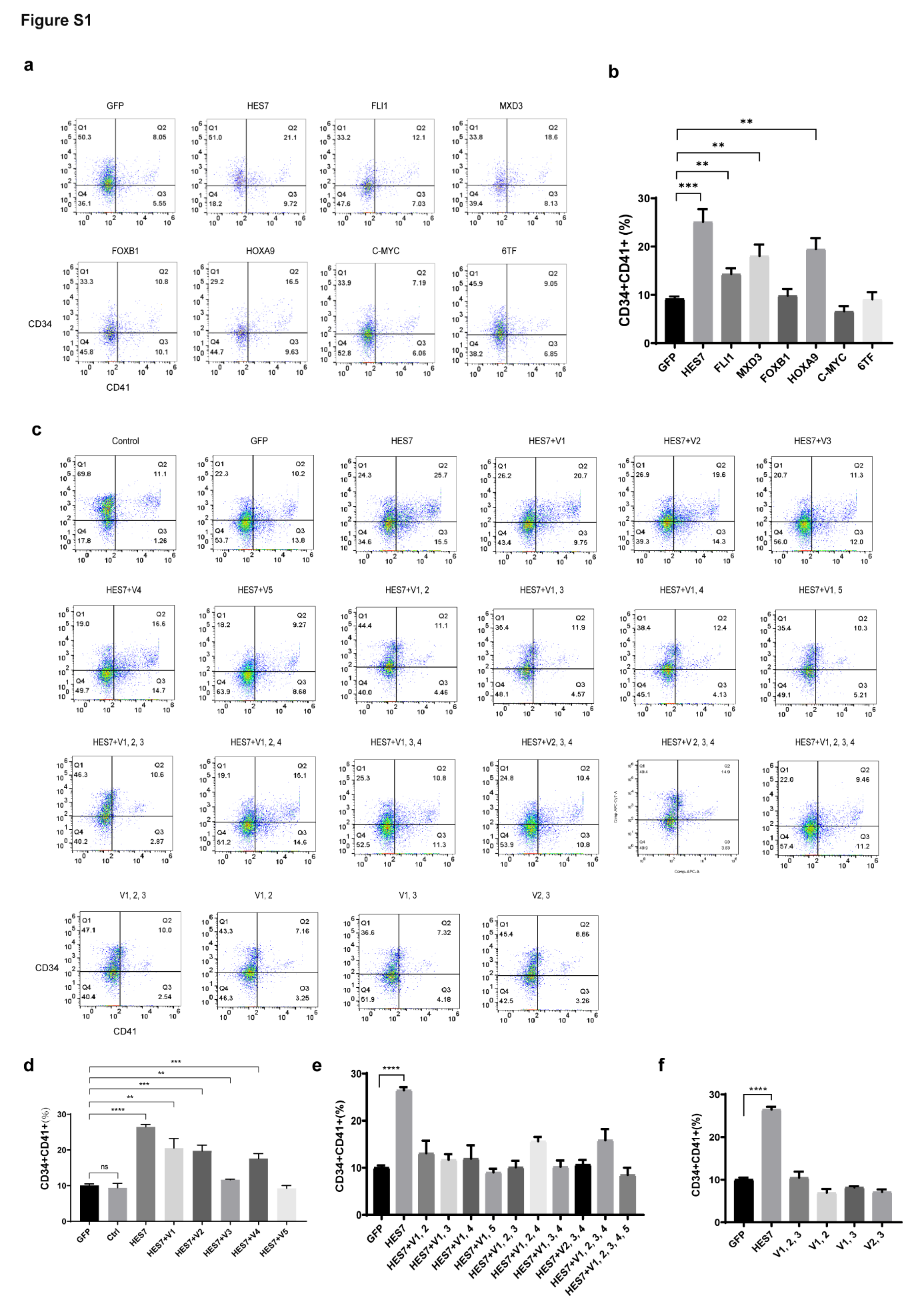


**Figure S1.**

1. Flow cytometry analysis of single-factor overexpression on MkP differentiation on Day 10.
2. Quantification of CD34^+^CD41^+^ cell populations in (a).

(c) Evaluation of the effect of different TF overexpressing on MkP differentiation. V1: FLI1, V2: MXD3, V3: FOXB1, V4: HOXA9, V5: C-MYC.

(d-f) Quantification of CD34^+^CD41^+^ cell populations in (c).

Data are means ± SD of biologically independent samples. Statistical significance was calculated using an ordinary one-way ANOVA. The statistical significance indicated in the figures was assigned as **p < 0.01; ***p < 0.001; ****p <0.0001.


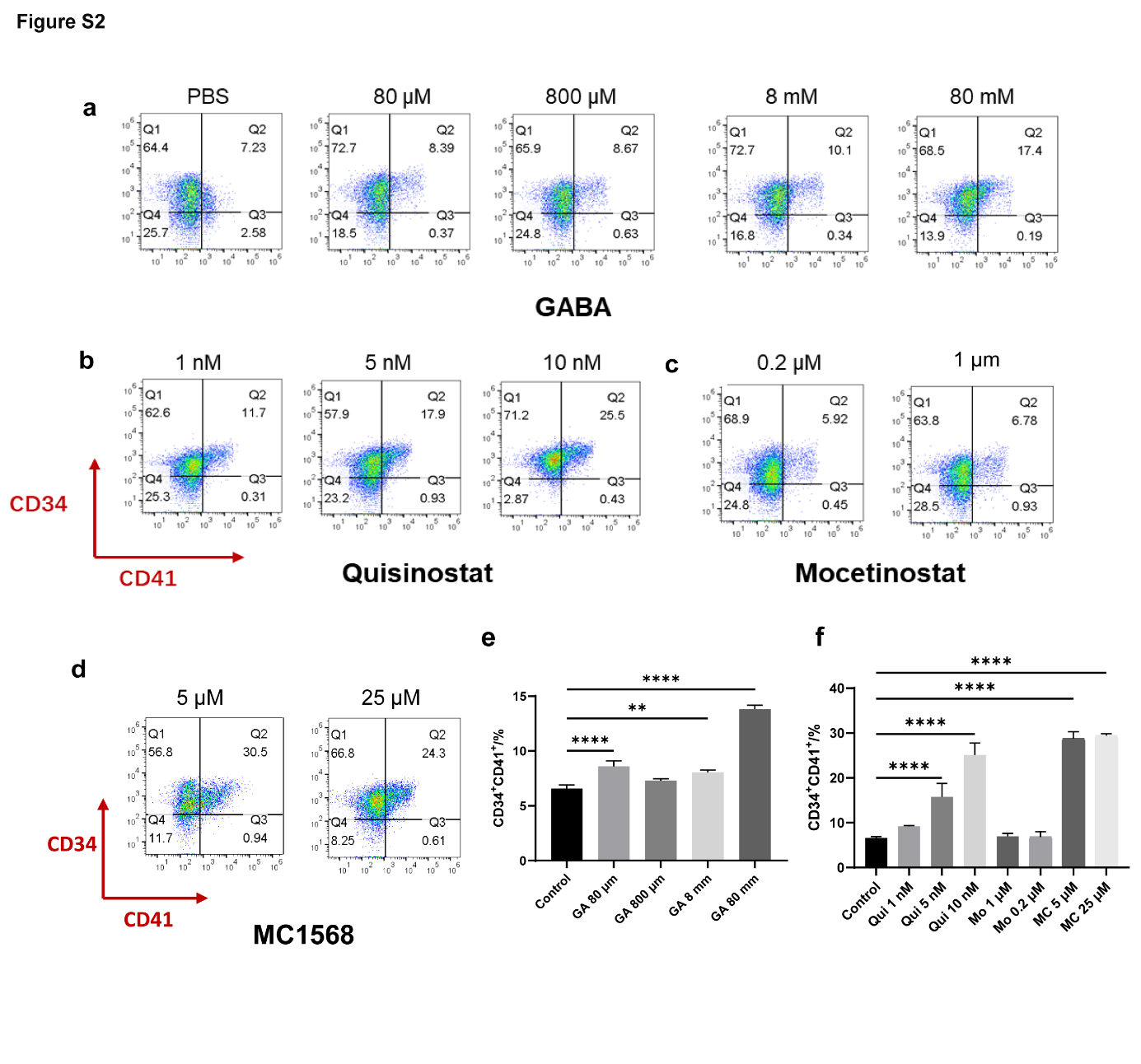


**Figure S2.**

(a-d) Flow cytometry analysis of CD34^+^ CD41^+^ cell populations treated for 7 days with GABA (80 µM, 800 µM, 8 mM, 80 mM), Quisinostat (1 nM, 5 nM, 10 nM), Mocetinostat (0.2 µM, 1 µM), and MC1568 (5 μM, 25 μM).

(e-f) Quantification of CD34^+^CD41^+^ cell populations in (a-d).

Data are means ± SD of biologically independent samples. Statistical significance was calculated using an ordinary one-way ANOVA. The statistical significance indicated in the figures was assigned as **p < 0.01; ****p <0.0001.


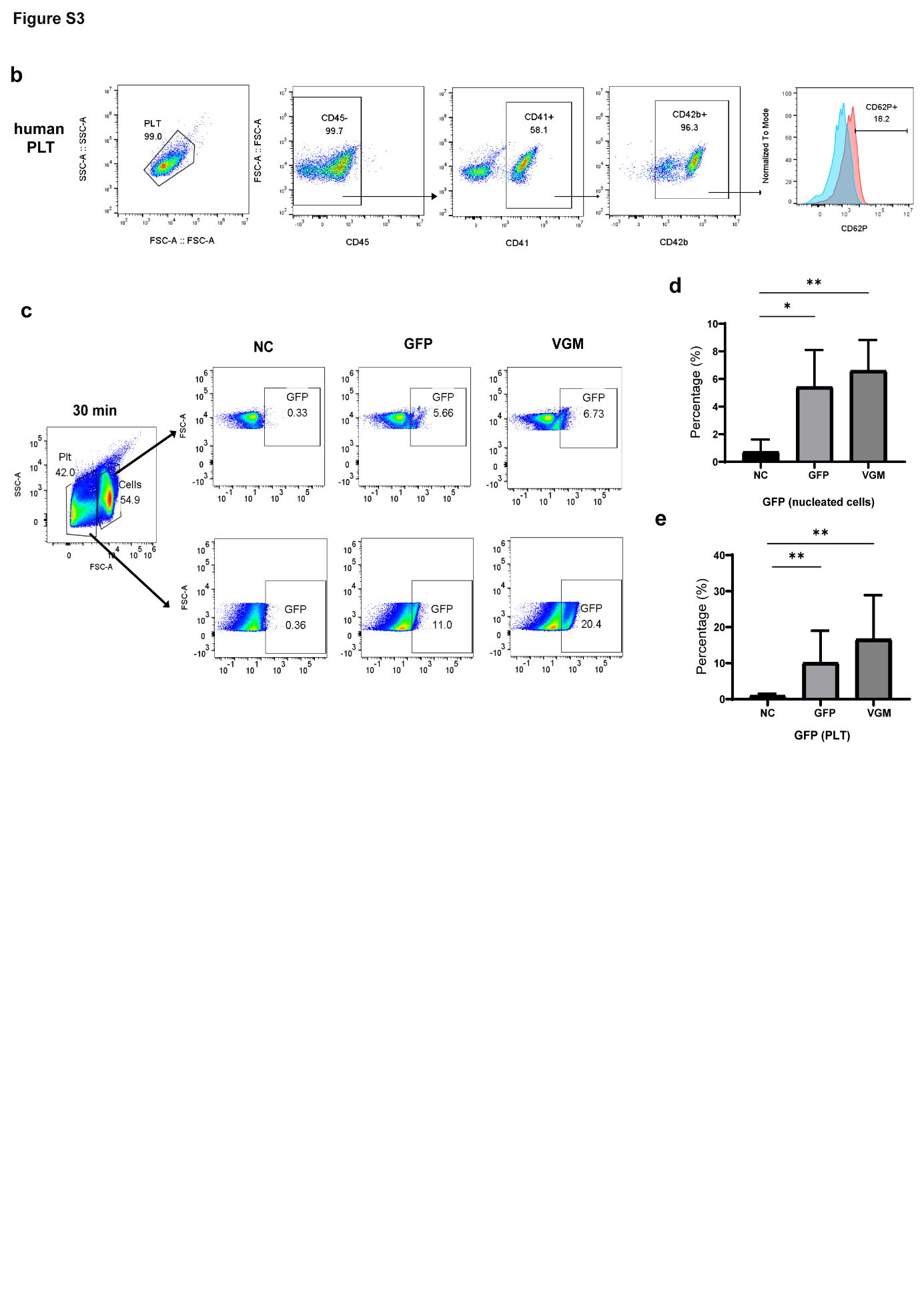


**Figure S3.**

(a) Flow cytometry analysis of platelet populations (CD45^−^CD41^+^CD42b^+^) of platelet from human whole blood. Platelets were activated with ADP and TRAP-6. Blue, unactivated platelets; red, activated platelets.

(b) Flow cytometry analysis of GFP expression in nucleated cells and platelets from tail vein blood at 30 min post-injection in mice.

(c-d) Statistical chart showing GFP expression in nucleated cells (b) and platelets (c) from tail vein blood at 30 min post-injection in mice (n=5).

Data are means ± SD of biologically independent samples. Statistical significance was calculated using a two-tailed t-test and an ordinary one-way ANOVA. The statistical significance indicated in the figures was assigned as *p < 0.05; **p < 0.01.


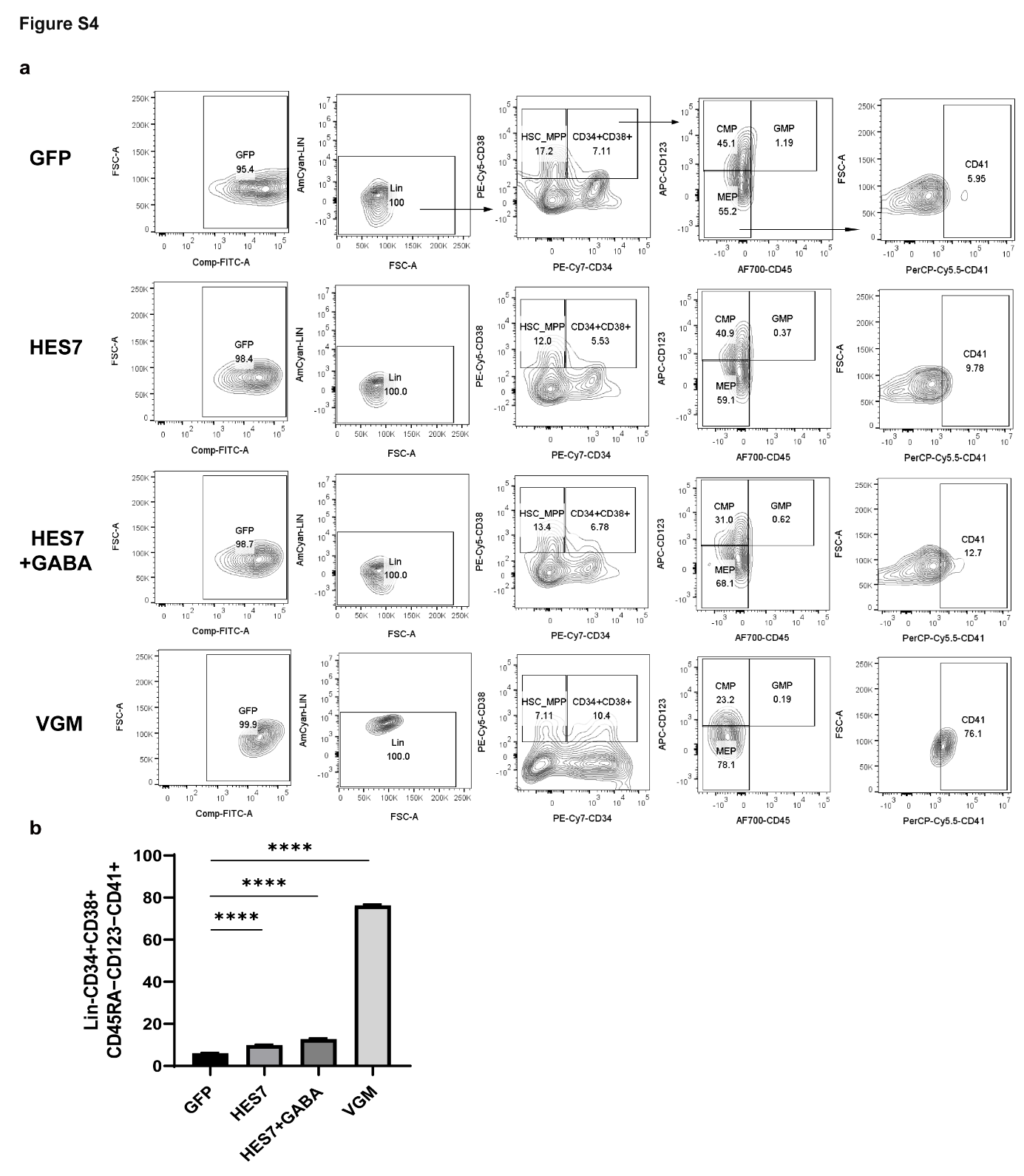


**Figure S4.**

(a) The flow cytometry results delineate the gating strategy for isolating MkPs from differentiated cell populations after 10 days of Stage 1 treatment. The analysis specifically utilizes markers including Lin^−^, CD34^+^, CD38^+^, CD45RA^−^, CD123^−^, and CD41^+^ to identify and isolate MkPs following treatments with GFP alone, HES7 alone, HES7 combined with GABA, and VGM.

(b) Quantification of CD34^+^CD41^+^ cell populations in (a).

Data are means ± SD of biologically independent samples. Statistical significance was calculated using an ordinary one-way ANOVA. The statistical significance indicated in the figures was assigned as ****p <0.0001.
